# Peripheral Blood Mononuclear Cells and Serum Cytokines in Patients with Lupus Nephritis after COVID-19

**DOI:** 10.3390/ijms25158278

**Published:** 2024-07-29

**Authors:** Katarzyna A. Lisowska, Klaudia Ciesielska-Figlon, Michał Komorniczak, Barbara Bułło-Piontecka, Alicja Dębska-Ślizień, Anna Wardowska

**Affiliations:** 1 Department of Pathophysiology, Faculty of Medicine, Medical University of Gdańsk, 80-211 Gdańsk, Poland; katarzyna.lisowska@gumed.edu.pl (K.A.L.);; 2 Department of Nephrology, Transplantology and Internal Medicine, Faculty of Medicine, Medical University of Gdańsk, 80-211 Gdańsk, Poland

**Keywords:** T cells, B cells, monocytes, dendritic cells, cytokines, lupus nephritis, SARS-CoV-2, COVID-19

## Abstract

Systemic lupus erythematosus (SLE) patients have an increased risk of infections and infection-related mortality. Therefore, during the global SARS-CoV-2 pandemic, SLE patients were particularly vulnerable to SARS-CoV-2 infections. Also, compared to other patients, SLE patients seem to develop more severe manifestations of coronavirus disease 2019 (COVID-19), with higher rates of hospitalization, invasive ventilation requirements, or death. This study evaluated the immune parameters after SARS-CoV-2 infection in SLE patients. We analyzed subpopulations of peripheral blood cells collected from patients with renal manifestation of SLE (lupus nephritis, LN). LN patients were divided into two subgroups: those unexposed to SARS-CoV-2 (LN CoV-2(−)) and those who had confirmed COVID-19 (LN-CoV-2(+)) six months earlier. We analyzed basic subpopulations of T cells, B cells, monocytes, dendritic cells (DCs), and serum cytokines using flow cytometry. All collected data were compared to a healthy control group without SARS-CoV-2 infection in medical history. LN patients were characterized by a decreased percentage of helper T (Th) cells and an increased percentage of cytotoxic T (Tc) cells regardless of SARS-CoV-2 infection. LN CoV-2(+) patients had a higher percentage of regulatory T cells (Tregs) and plasmablasts (PBs) and a lower percentage of non-switched memory (NSM) B cells compared to LN CoV-2(−) patients or healthy controls (HC CoV-2(−)). LN patients had a higher percentage of total monocytes compared with HC CoV-2(−). LN CoV-2(+) patients had a higher percentage of classical and intermediate monocytes than LN CoV-2(−) patients and HC CoV-2(−). LN CoV-2(+) patients had higher serum IL-6 levels than HC CoV-2(−), while LN CoV-2(−) patients had higher levels of serum IL-10. LN patients are characterized by disturbances in the blood’s basic immunological parameters. However, SARS-CoV-2 infection influences B-cell and monocyte compartments.

## 1. Introduction

The search for factors causing the breakdown of tolerance and the induction of autoimmune processes in predisposed individuals showed the influence of several environmental and infectious elements [1]. The problem of infections seems especially important in the pathogenesis of autoimmune disease. Immune responses against viral or bacterial antigens may cause disease if the reaction is excessive or the microorganisms resist eradication. In addition, viral infections can contribute to the formation of immune complexes, which deposit in organs and tissues, triggering inflammation. Sometimes, antibodies against microbes cross-react with self-antigens. The process is associated with molecular mimicry, i.e., the similarity between antigens of microorganisms and autoantigens present in the human body. Furthermore, disorders of programmed cell death, i.e., apoptosis, especially impairment in dead cell clearance, promote the phenomenon of “bystander activation” [2,3]. In the case of an already-developed autoimmune disease, each viral infection exacerbates the symptoms of the underlying disease [4].

Infections cause one-third of all hospitalizations and deaths among systemic lupus erythematosus (SLE) patients [5]. The incidence of coronavirus disease 2019 (COVID-19) infection may affect as many as 18.1% of SLE patients, and the hospitalization rates range from 0.24% to 10.6% [6]. A meta-analysis by Yurkovich et al. [7] showed that mortality caused by an infectious agent is five times higher in SLE patients than in the general population. Increased mortality due to infection is also associated with specific changes in the immune system, causing a higher incidence of infectious diseases in SLE patients. These elements include reduced levels of receptors for the complement system (CR1, CR2, CR3) on immunocompetent cells, deficiencies of receptors involved in the removal of pathogens, e.g., mannose-binding lectin, or gene polymorphism for immunoglobulin Fc receptors [8]. Disturbances in the function and activation of peripheral blood cells, including abnormalities in chemotaxis, phagocytosis, the production of reactive oxygen species, or the secretion of cytokines, are associated with a weaker response to pathogens [1]. Also, the therapy routinely used in SLE patients increases the risk of severe viral infections, including SARS-CoV-2 [9,10]. Our knowledge about COVID-19 in patients with SLE is based on central studies describing the environmental and social conditions and phenomena accompanying SARS-CoV-2 infections [11,12]. What we know is that, compared to patients with other autoimmune diseases, SLE patients seem to develop more severe manifestations of COVID-19 infection, with higher rates of hospitalization, invasive ventilation requirements, or death [13], which may be related to severe impairment of the immune response at many levels, a genetic predisposition, or chronic immunosuppressive treatment.

From the immunological point of view, all immune mechanisms, innate and acquired, are involved in the fight against SARS-CoV-2. However, the response of cells of adaptive immunity, T and B cells, seems to be the most important for COVID-19 infection. Studies show that severe COVID-19 infection can be associated with a striking loss of B cells [14] and T cells [14,15,16]. Some authors show that patients with COVID-19 pneumonia are characterized by decreased naive, central memory (Tcm cells), or effector memory T cells (Tem cells) [15], while others saw no changes in the T-cell memory compartment [16]. Studies show that T cells of COVID-19 patients express high levels of activation markers such as CD25 [17], HLA-DR, and CD69, even during the convalescent phase [18]. Many studies have also demonstrated that T cells of COVID-19 patients present an exhausted or senescent phenotype [19,20]. As for B cells, Mathew et al. [14] showed that patients with severe COVID-19 have decreased frequencies of class-switched (SM) and non-switched (NSM) memory B cells compared with healthy people or recovered donors.

De Biasi et al. [15] showed that patients with severe COVID-19 have higher plasma levels of several cytokines and chemokines, including tumor necrosis factor (TNF), interferon-gamma (IFN-γ), interleukin 1 alpha (IL-1α), IL-1β, IL-4, IL-6, IL-7, IL-8, IL-10, CCL2, or CCL3. CCL2 is known as a potent chemokine recruiting monocytes. Some studies show that severe COVID-19 is accompanied by a decreased percentage of blood monocytes [16,21], especially in non-classical populations [22]. Some authors also reported decreased percentages of dendritic cells (DCs) in the peripheral blood of COVID-19 patients in the acute or convalescent phase [23].

While many of the reported immune changes in new COVID-19 infections result from an immune response to the virus, little is known about how long these changes last. Compared to other viruses in this group, SARS-CoV-2 is less virulent but much more infectious. What is more, even people who recover from mild infection may continue to experience symptoms such as brain fog, headaches, dizziness, depression, and anxiety, as well as cardiac arrhythmias, chest pains, and mental disturbances for many weeks or even months. A study by Taeschler et al. [24] revealed that T-cell count in the peripheral blood of patients with severe COVID-19 returned to normal six months after infection and remained stable for the next six months. However, T cells of recovered patients were still characterized by increased expression of activation markers CD38 and HLA-DR, which correlated with the level of IL-6 and TNF-α 6 and 12 months after infection. Govender et al. [25] showed a significant decrease in the percentage of CD4+ T cells after 6 weeks and 6–7 months post-infection, with a simultaneous increase in CD8+ T cells. The authors also saw increased CD4+ Tcm cells over time, with no alterations in the CD4+ Temra or Tem cells. At the same time, the percentage of naive CD8+ T cells increased while CD8+ Temra cells decreased with time.

Thus far, only one study demonstrated changes in T-cell and B-cell subpopulations of COVID-19-convalescent SLE [26]. The authors showed that patients 12 weeks after infection displayed a reduction in CD8+ T cells compared to unexposed patients, especially in the naive compartment. At the same time, they had an increased percentage of memory CD8+ T cells. They did not observe any changes in CD4+ T-cell subpopulations compared with unexposed patients. COVID-19-convalescent patients also had a decreased percentage of naive B cells and an increased frequency of memory B cells and plasmablasts (PBs).

Due to the lack of articles in the literature describing the behavior of the immune system of SLE patients in response to SARS-CoV-2 infection, the presented study aimed to evaluate the immune system-affecting outcomes of SARS-CoV-2 infection in SLE patients. In order to achieve this goal, we compared basic subpopulations of T and B cells, monocytes, dendritic cells, and serum cytokine levels between patients with the renal manifestation of SLE (lupus nephritis, LN) who had an infection six months earlier, patients unexposed to SARS-CoV-2, and healthy people also unexposed to the virus. We focused on the expression of selected activation antigens and memory compartments of immune cells.

## 2. Results

### 2.1. Characteristics of Patients

The study groups consisted of 13 healthy women who had not been diagnosed with SARS-CoV-2 infection in the last 12 months (HC CoV(−)), 17 female LN patients who had COVID-19 with laboratory-confirmed SARS-CoV-2 infection 6 months earlier (LN CoV-2(+)), and 10 female LN patients unexposed to SARS-CoV-2 (LN CoV-2(−)) infection in the last 12 months (Table 1).

Patients fulfilled the Systemic Lupus International Collaborating Clinics classification criteria (SLICC). SLE activity was assessed using the SLE Disease Activity Index (SLEDAI) and the Systemic Lupus Activity Measure—revised (SLAM-R). The results obtained for all scales are presented in Table 1. There was no significant difference between patients regarding the duration of SLE and LN (Table 1). No difference was seen in eGFR (estimated glomerular filtration rate), serum ESR (erythrocyte sedimentation rate), or anti-dsDNA (anti-double-stranded DNA) between LN patients.

During the SARS-CoV-2 infection, most LN patients complained of fever, chills, cough, fatigue, muscle or body aches, and a runny nose. Only three patients required hospitalization. None of the patients developed long COVID or post-COVID.

### 2.2. Changes in T-Cell and B-Cell Subpopulations

**Figure 1:**
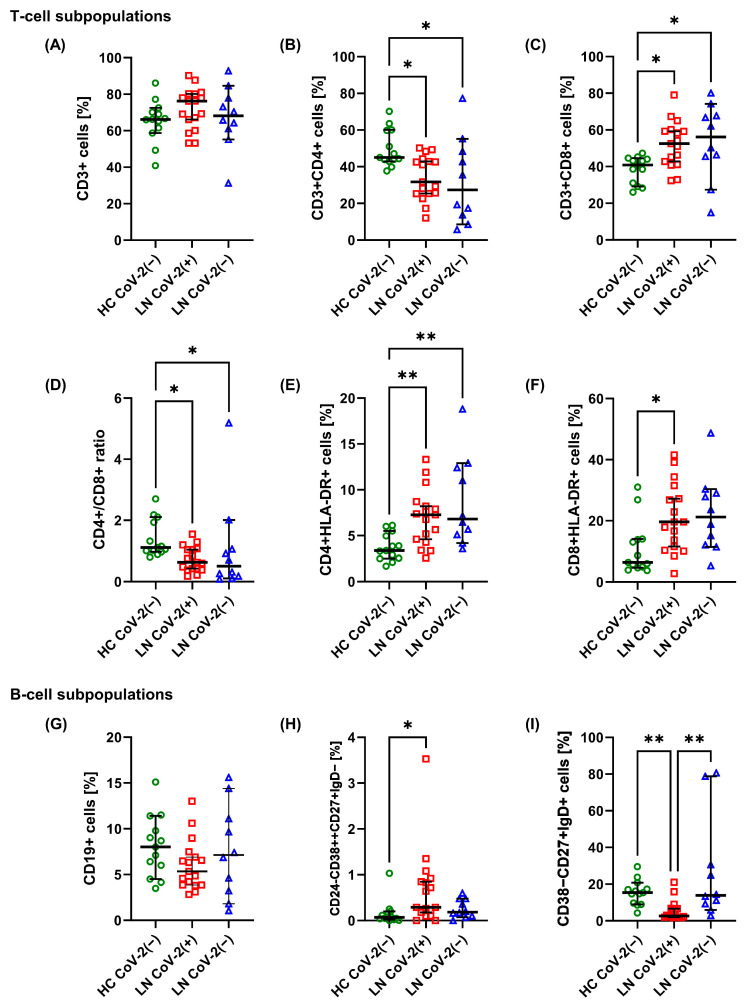
Comparison of basic T-cell and B-cell subpopulations. Graphs show the percentage of T (CD3+) cells, (**A**), helper (CD3+CD4+) T cells (**B**), cytotoxic (CD3+CD8+) T cells (**C**), CD4+/CD8+ ratio (**D**), activated (with HLA-DR expression) helper T cells (**E**), activated cytotoxic T cells (**F**), B (CD19+) cells (**G**), plasmablasts (PBs) (**H**) and non-switched memory (NSM) B cells (**I**) in healthy control (HC CoV-2(−)), LN patients six months after SARS-CoV-2 infection (LN CoV-2(+)), and LN patients unexposed to SARS-CoV-2 (LN CoV-2(−)). Data are presented as median with 95% CI according to the Kruskal–Wallis test with a post hoc Dunn test, green circles, red squares, and blue rectangles represent individual results, * *p* < 0.05, ** *p* < 0.01.

There was no difference in the percentage of CD3+ T cells (Figure 1A) between healthy control (HC) and LN patients. LN CoV-2(+) and LN CoV-2(−) patients were characterized by a decreased percentage of helper (CD3+CD4+) T (Th) cells (Figure 1B) and CD4+/CD8+ ratio (Figure 1D) compared with HC with a simultaneous increase in the percentage of cytotoxic (CD3+CD8+) T (Tc) cells (Figure 1C). T-cell proportions did not differ between LN patients with or without SARS-CoV-2 infection. Within Th and Tc cells, we further identified specific cell subpopulations. And so, LN CoV-2(−) patients were also characterized by a decreased percentage of CD4+CD28+ cells compared with HC CoV(−) with an unaltered percentage of CD8+CD28+ cells (Table 2). The percentage of CD69+ cells was comparable between the groups in both tested T-cell populations (Table 2). Also, LN CoV-2(+) and LN CoV-2(−) patients had a higher percentage of CD4+HLA-DR+ (Figure 1E). Meanwhile, the percentage of CD8+HLA-DR+ was increased only in LN CoV-2(+) patients compared to HC CoV(−) (Figure 1F).

The analysis of surface expression of CD197 (CCR7) and CD45RA within CD4-positive and CD8-positive subpopulations allowed us to follow the phenotypic classification of the T-cell memory compartment: naive T cells (CD197+CD45RA+ cells), central memory T cells (Tcms) (CD197+CD45RA− cells), effector memory T cells (Tems) (CD197−CD45RA− cells), and effector memory re-expressing T cells (Temras) (CD197−CD45RA+ cells). There was no difference in the percentage of CD4+ naïve T cells, CD4+ Tcm, CD4+ Tem, and CD4+ Temra cells (Table 2). LN CoV-2(−) patients had a lower percentage of CD8+ Tcm cells compared with HC CoV(−) (Table 2). No changes were observed in CD8+ naïve T cells, CD8+ Tem, and CD8+ Temra cells (Table 2). However, LN CoV-2(+) patients were characterized by an increased percentage of Tregs (CD4+CD127−CD25+ cells) compared with HC CoV(−) (Table 2).

We also identified transitional B cells (TBs), plasmablasts (PBs), double-negative memory (DNM) B cells, switched memory (SM) B cells, non-switched memory (NSM) B cells, and naive B cells. There was no difference in the percentage of B (CD19+) cells (Figure 1G) between HC and both LN groups. No changes were observed in the percentage of TBs (CD24++CD38++), naïve (CD38−CD27−IgD+) B cells, DNM (CD38−CD27−IgD−) B cells, or SM (CD38−CD27+IgD−) B cells (Table 2). However, LN CoV-2(+) patients had a higher percentage of PBs (CD24−CD38++CD27+IgD−) compared to HC (Figure 1H). At the same time, they had a lower percentage of NSM (CD38−CD27+IgD+) B cells compared to LN CoV-2(−) patients and HC (Figure 1I).

### 2.3. Changes in Monocyte and DC Subpopulations

**Figure 2:**
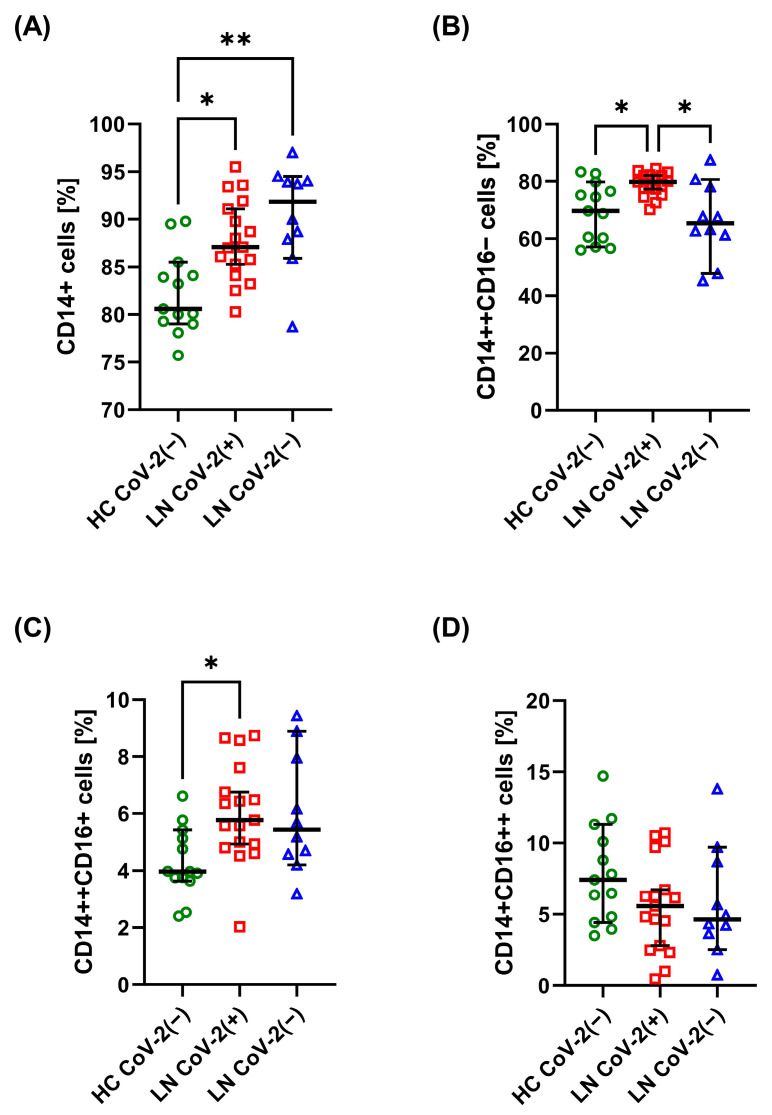
Comparison of basic monocyte subpopulations. Graphs show the percentage of total (CD14+) monocytes (**A**), classical (CD14++CD16−) monocytes (**B**), intermediate (CD14++CD16+) monocytes (**C**), and non-classical (CD14+CD16++) monocytes (**D**) in healthy control (HC CoV-2(−)), LN patients six months after SARS-CoV-2 infection (LN CoV-2(+)), and LN patients unexposed to SARS-CoV-2 (LN CoV-2(−)). Data are presented as median with 95% CI according to the Kruskal–Wallis test with a post hoc Dunn test, green circles, red squares, and blue rectangles represent individual results, * *p* < 0.05, ** *p* < 0.01.

LN CoV-2(+) and LN CoV-2(−) patients had a higher percentage of total monocytes compared with HC CoV(−) (Figure 2A). LN CoV-2(+) had a higher percentage of classical monocytes (CD14++CD16−) compared with LN CoV-2(−) patients and HC CoV(−) (Figure 2B). They also had a higher percentage of intermediate monocytes (CD14++CD16+) compared with HC CoV(−) (Figure 2C). No difference was observed in the percentage of non-classical monocytes (CD14+CD16++) (Figure 2D). LN CoV-2(−) patients had a significantly lower percentage of classical monocytes with TLR2 expression compared with HC CoV(−). In comparison, LN CoV-2(+) had a higher percentage of classical monocytes with TLR4 expression compared with HC CoV(−) (Table 3). LN CoV-2(+) patients had a significantly lower percentage of intermediate monocytes with TLR2 expression compared with HC CoV(−). No difference was observed in the percentage of any subclass of monocytes in terms of expression of CD162, CX3CR1, or CCR2.

Among dendritic cells (DCs), we analyzed the percentages of conventional dendritic cell type 1 (cDC1), conventional dendritic cell type 2 (cDC2), DC2, DC3, and inflammatory DC3. No differences were observed in the percentage of any of the DC subpopulations, including cDC1 (Clec9A+ cells), cDC2 (CD1c+FcεRIα+ cells), DC2 (CD5+CD163− cells), DC3 (CD5−CD163+ cells), inflammatory DC3 (CD14+CD163+ cells), or CD11c cells with expression of CD80 and CD86 (Table 4).

### 2.4. Changes in Serum Cytokines, SARS-CoV-2 Spike Ig, and Nucleocapsid

**Figure 3:**
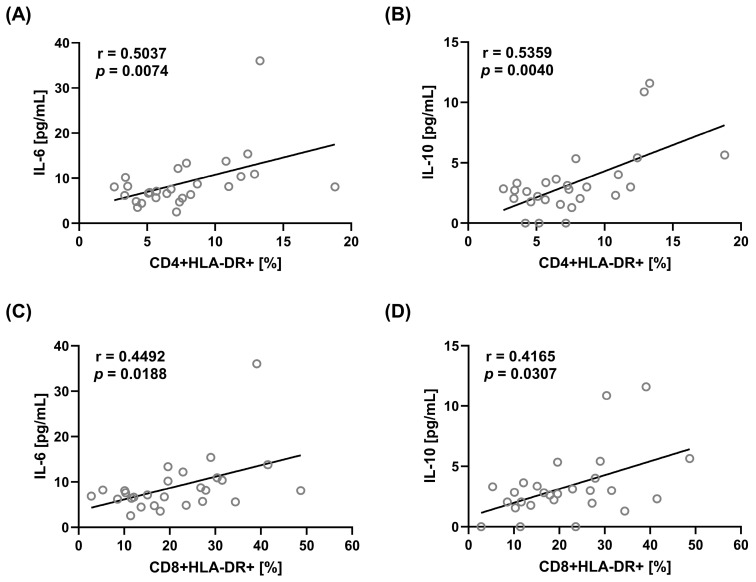
Correlations between percentages of T-cell subpopulations with HLA-DR expression and serum IL-6 and IL-10 in LN patients. Graphs show correlations between CD4+HLA-DR+ cells and IL-6 (**A**) and IL-10 (**B**), CD8+HLA-DR+ and IL-6 (**C**), and IL-10 (**D**) cells, Spearman Rank Correlation test, *p* < 0.05.

There were changes in serum cytokine levels for two of seven tested cytokines, IL-6 and IL-10 (Table 5). Serum IL-10 concentration was significantly higher in the LN CoV-2(−) patients compared with the HC. Serum IL-6 was significantly higher in LN CoV-2(+) patients than in HC (Table 5). Serum IL-6 was positively correlated with the percentage of CD4+HLA-DR+ cells (Figure 3A) and CD8+HLA-DR+ (Figure 3C). Also, IL-10 was positively correlated with the percentage of CD4+HLA-DR+ cells (Figure 3B) and CD8+HLA-DR+ (Figure 3D). There was also a positive correlation between serum IL-6 and erythrocyte sedimentation rate (ESR) (r = 0.4038, *p* = 0.0408).

There was no difference in the serum IFN-γ, IL-2, IL-4, IL-17A, or TNF between the studied groups. All patients and healthy individuals presented high serum Spike Ig and barely detectable Nucleocapsid. No difference was observed in the serum Spike Ig and Nucleocapsid between study groups (Supplementary Table S1).

## 3. Discussion

In this paper, we aimed to evaluate whether SARS-CoV-2 infection caused long-term changes in the immune system of LN patients. Therefore, we compared basic subpopulations of T and B cells, monocytes, dendritic cells, and serum cytokine levels between LN patients who had an infection six months earlier (LN CoV-2(+)) with patients unexposed to SARS-CoV-2 (LN CoV-2(−)) and healthy people (HC CoV-2(−)) also unexposed to the virus. All individuals who qualified for the study were vaccinated against SARS-CoV-2. Moreover, they all presented high serum Spike Ig and barely detectable Nucleocapsid.

Although the number of research articles raising the issue of the immune system changes in patients infected with SARS-CoV-2 is relatively high, the inconsistency in study group selection may hamper meaningful conclusions. Many of the first articles focused primarily on patients who developed severe complications related to the cytokine storm during COVID-19 [16,21]. Then, articles describing changes in some immunological parameters in patients with mild or moderate COVID-19 began to appear [14,15]. An incredible advantage of these publications was the assessment of changes during infection. However, one of a few publications focused on the possible persistence of the immunological changes 3, 6, and 12 months after infection in individuals with no autoimmune disease in their medical history. This is justified by the fact that many people have persistent symptoms after infection in the form of long COVID or post-COVID. Furthermore, only one study demonstrated changes in T-cell and B-cell subpopulations of COVID-19-convalescent SLE [26].

Many of the changes we observed in T- and B-cell subpopulations were unrelated to SARS-CoV-2 infection. These seemed to be associated with the autoimmune disease itself. Such parameters included decreased percentages of CD3+CD4+ (Th) cells and increased percentages of CD3+CD8+ (Tc) cells in LN patients regardless of COVID-19 status. The presence of HLA-DR characterized both T-cell subpopulations and was associated with higher serum IL-6 and IL-10, suggesting that the activation of T cells accompanies the disease. Similar changes in T cells have already been described in SLE patients, especially in those with renal involvement [27,28]. In COVID-19 patients with no history of SLE, increased percentages of T cells with HLA-DR expression were observed during infection [14,15], and this persistent T-cell activation continued even twelve months later [24]. Therefore, one could assume that the increased activity of T cells in LN patients does not have to be related to COVID-19.

LN patients who underwent SARS-CoV-2 infection six months before blood sampling also presented several specific alterations, including increased PBs and decreased NSM B cells. Similar changes were reported in COVID-19 patients during infection [14,29] and SLE patients three months after infection [26]. Plasmablasts arise from B cells early during an infection and are capable of proliferating and producing antibodies. The increase in PB percentage is mainly associated with the acute phase of COVID-19 [29,30]. Nevertheless, it is also associated with a higher survival rate [31]. According to several articles, plasmablasts return to baseline levels in convalescent patients within six months after infection [29]. However, some authors demonstrated that these cells can still be high a few months later [26], which could be associated with specific long-lived plasma cells that are still detectable in the bone marrow of convalescent patients even 7–8 months after infection [32]. These data support the thesis that SARS-CoV-2 infection stimulates a robust, durable humoral response months later. It should be emphasized that the observed increase in PBs is not related to repeated vaccinations because all patients qualified for the study were vaccinated against SARS-CoV-2 several times, and changes in PBs are limited only to patients who underwent infection.

LN patients after COVID-19 also had an increased percentage of Tregs responsible for suppressing and regulating immune responses. According to some studies, the percentage of Tregs rises during COVID-19 [15,33]. Moreover, their highest values were reported in patients in the most critical conditions, requiring intubation or extracorporeal membrane oxygenation (ECMO) [33]. However, some studies show that patients with severe COVID-19 have reduced Treg numbers compared with patients with mild infection [34]. Several studies examined changes in Tregs in recovered patients or patients with long COVID-19 with contradictory findings [35,36]. According to Galán et al. [35], long COVID is associated with higher Treg percentages, while Utrero-Rico et al. [36] demonstrated that patients with persistent symptoms have lower Treg percentages than recovered patients. Unfortunately, analysis of Treg numbers or frequencies alone cannot establish their function in people with SARS-CoV-2 infection. Therefore, additional studies investigating the immunosuppressive activities of Tregs are highly recommended.

Dendritic cells, macrophages, and monocytes form the mononuclear phagocytic system (MPS) and are professional antigen-presenting cells (APCs). In addition, the role of classical and intermediate monocytes is to control infection and the inflammation associated with it. Under pathological conditions, like viral or bacterial infections, monocytes are activated and recruited by inflammatory mediators to infiltrate affected tissue. In SLE, they play a significant role as cells responsible for presenting nuclear antigens to CD4+ T cells and initiating a process that leads to pathological production of ANAs (anti-nuclear antibodies). In COVID-19, dysregulation of monocytes and macrophages is associated with cytokine storm.

Research by other authors shows that patients with moderate SARS-CoV-2 infection are characterized by decreased percentages of non-classical monocytes and increased intermediate monocytes [22]. The inflammatory storm in COVID-19 is driven by CD14+CD16+ monocytes expressing IL-6, as demonstrated in patients in intensive care units [21]. Finally, recently recovered patients and patients with long COVID have higher percentages of classical and intermediate monocytes than individuals not exposed to the virus [37]. In our study, we could not detect any specific changes in DCs. LN patients were characterized by an increased percentage of monocytes compared with healthy individuals regardless of COVID-19 infection. However, recovered patients had a higher percentage of classical and intermediate monocytes. They also had a higher percentage of classical monocytes with TLR4 expression and increased serum IL-6. Of 17 LN patients, only 3 required hospitalization. Also, none of the patients developed long COVID or post-COVID. Therefore, it seems that monocyte activation is not only important for the development of COVID-19 symptoms. It also persists several months after the infection has passed without any complications.

As for DCs in COVID-19, several authors demonstrated that these percentages are decreased in COVID-19 patients in the acute or convalescent phase [23,38]. Firstly, it should be noted that DCs constitute a heterogeneous group of cells of various origins. In humans, the basic division distinguishes between plasmacytoid DCs (pDCs) and conventional DCs (cDCs) [39]. pDCs morphologically resemble plasma cells and are mediators of antiviral immunity through the ability to produce type I interferons (IFNs) [40]. Meanwhile, cDCs present antigens to T cells and cDC1s stimulate CD8+ T cells, whereas cDC2s activate CD4+ T cells [41]. Further, cDC2s can be divided into DC2 and DC3. Pérez-Gómez et al. [38] demonstrated that COVID-19 patients have significantly decreased total percentages of cDCs and cDC2. The authors also saw a decrease in pDCs. Seven months later, patients still displayed a decrease in cDCs and pDC2. Similar data were demonstrated by Zhou Y et al. [21]. We focused mainly on subpopulations of cDC, and we did not see any changes in LN patients regardless of COVID-19 infection. In our opinion, the main reason for the different results is the study group because SLE patients differ significantly from healthy people in terms of DC numbers and activity. This means that they may react differently to viral infections such as SARS-CoV-2. While the authors cannot agree on the numerical composition of DCs in patients’ blood, it is established that insufficient clearance of dying cells and increased activity of DCs are responsible for priming autoimmune T- and B-cell reactivity [42].

Our study has several limitations. The study groups are relatively small, especially the LN-CoV-2(−) group. As mentioned in the Introduction, SLE patients are particularly vulnerable to viral and bacterial infections. Most of the patients under the care of the Department of Nephrology, Transplantology, and Internal Disease experienced SARS-CoV-2 infection. Only ten patients who did not become ill were identified. Another problem is the lack of results of immunological tests in the LN CoV-2(+) group before the infection. This, in turn, results from research funding being obtained after the end of the COVID-19 pandemic. Most patients had had the infection one or two years earlier. We only managed to find seventeen patients who had fallen ill six months earlier. An incredible advantage of our study is the uniformity of the study groups. SLE mainly affects women, so only women were included in the study. Furthermore, all patients suffered from LN. All women participating in the study were vaccinated. Moreover, unlike Sole et al. [26], we included healthy individuals in our study to determine the immunological status of SLE patients before the infection.

## 4. Materials and Methods

### 4.1. Study Groups

The study groups consisted of 17 female LN patients who had COVID-19 with laboratory-confirmed SARS-CoV-2 infection 6 months earlier (LN CoV-2(+)), 10 female LN patients unexposed to SARS-CoV-2 infection in the last 12 months (LN CoV-2(−)), and 13 healthy women, who had not been diagnosed with SARS-CoV-2 infection in the last 12 months (HC CoV(−)) (Table 1). All patients and healthy people were vaccinated against SARS-CoV-2. All LN patients were recruited at the Department of Nephrology, Transplantology, and Internal Medicine at the Medical University of Gdańsk.

Patients fulfilled the Systemic Lupus International Collaborating Clinics classification criteria (SLICC). SLE activity was assessed using the SLE Disease Activity Index (SLEDAI) and the Systemic Lupus Activity Measure-revised (SLAM-R).

The tested material was peripheral venous blood collected into EDTA tubes to determine T-cell, B-cell, and DC subpopulations and 5 mL of blood collected in heparin tubes to analyze monocyte subpopulations. In addition, 5 mL of blood was collected into anticoagulant-free tubes to collect serum to assess concentrations of cytokines, SARS-CoV-2 Spike Ig, and Nucleocapsid. We stored serum samples at 80 °C.

### 4.2. Determination of T-Cell, B-Cell, and Monocyte Subpopulations Ex Vivo

In total, 100 μL of blood sample per tube was transferred for staining with monoclonal antibodies and red blood cell (RBC) lysis. First, RBCs were lysed with buffer. Cells were then washed with PBS (phosphate-buffered saline) and stained with antibodies against CD3 FITC, CD24 FITC, CD69 PE, CCR7 (CD197) PE, CD25 PE, IgD PE, CD28 APC, CD38 APC, CD8 APC-Cy7, CD4 V450, CD19 V450, HLA-DR V450, CD27 V500, HLA-DR V500 (BD Pharmingen™, San Diego, CA, USA), CD45RA APC, CD127 APC (BioLegend, San Diego, CA, USA) (staining panel for T and B cells), CD162 PE, TLR2 AF 647, CD16 APC-Cy7, HLA-DR V450, CD14 V500 (BD Pharmingen™, USA), CX3CR1 FITC, TLR4 PE, and CCR2 (CD192) APC (BioLegend, USA), (staining panel for monocytes) for 30 min at room temperature in the dark. After this time, cells were washed with PBS and suspended in 200 μL of suitable buffer for flow cytometric analysis using the FACSVerse instrument (Becton Dickinson, Franklin Lakes, NJ, USA).

### 4.3. Determination of DC Subpopulations in PBMCs

The peripheral blood mononuclear cells (PBMCs) were isolated from whole blood using density-gradient centrifugation on a Histopaque 1077 (Sigma-Aldrich Inc., Saint Louis, MO, USA). Cells were then washed with PBS and stained with antibodies against CD163 PE, CD80 PE, CD86 APC, CD14 APC-Cy7, CD5 V450, CD11c V450, HLA-DR V500 (BD Pharmingen™, USA), CD1c FITC, FcεR1α APC, and Clec9A PE (BioLegend, USA) for 30 min at 4 °C in the dark. Then, cells were washed with PBS and suspended in 200 mL of suitable buffer for flow cytometric analysis using the FACSVerse instrument (Becton Dickinson, Franklin Lakes, NJ, USA).

### 4.4. Cytokine Measurement in Serum Samples

A Cytometric Bead Array (CBA) Human Th1/Th2/Th17 Cytokines Kit (BD Biosciences, San Jose, CA, USA) was used according to the manufacturer’s protocol to determine the level of IL-2, IL-4, IL-6, IL-10, TNF, IFN-γ, and IL-17A in the serum samples from healthy people and both groups of patients. Quantitative cytometric fluorescence analysis was performed using the FACSAria III cytometer (Becton Dickinson, Franklin Lakes, NJ, USA). The detection range for all measured cytokines was between 20 and 5000 pg/mL. The kit performance was optimized to analyze physiologically relevant concentrations (pg/mL levels) of specific cytokine proteins in serum samples. The limit of detection for IL-2 was 2.6 pg/mL, IL-4 was 4.9 pg/mL, IL-6 was 2.4 pg/mL, IL-10 was 4.5 pg/mL, TNF was 3.8 pg/mL, IFN-γ was 3.7 pg/mL, and IL-17A was 8.9 pg/mL.

### 4.5. SARS-CoV-2 Spike Ig and Nucleocapsid Measurement in Serum Samples

A Human SARS-CoV-2 Spike (trimer) Ig ELISA kit (Invitrogen, Waltham, MA, USA) and a Human SARS-CoV-2 N ELISA kit (Invitrogen, Waltham, MA, USA) were used according to the manufacturer’s protocol to determine Spike Ig and Nucleocapsid levels. The minimum detectable dose of Spike Ig was 144 units/mL, and for Nuecleocapsid, it was 0.07 ng/mL. The density of color was proportional to the target amount of sample captured in the 96-well plate, and the O.D. absorbance was read at 450 nm absorbance using an Epoch™ Microplate Spectrophotometer (BioTek, Winooski, VT, USA).

### 4.6. Analysis and Statistics

The cytometric data were analyzed with the FlowJo 10 software (Beckton Dickinson, Ashland, USA). Thirty thousand events corresponding to viable lymphocytes’ light scatter characteristics were acquired from each sample to analyze T-cell subpopulations. First, lymphocytes were selected based on FSCs (forward scatter characteristics) and SSCs (side scatter characteristics) (Supplementary Figure S1A). Then, T cells were identified based on their posit,ivity for the CD3 antigen (Supplementary Figure S1B). Next, helper T (Th) cells were identified based on the expression of CD4 antigen and cytotoxic (Tc) T cells based on CD8 expression (Supplementary Figure S1C). Finally, subpopulations expressing different activation antigens, e.g., HLA-DR antigen (Supplementary Figure S1D–F), were identified within CD4-positive and CD8-positive subpopulations. Supplementary Figure S1D shows the percentage of CD8+HLA-DR+ cells in healthy controls (HC Co-V(−)), Supplementary Figure S1E shows the same value in LN patients six months after SARS-CoV-2 infection (LN CoV-2(+)), and Supplementary Figure S1F shows that in LN patients unexposed to SARS-CoV-2 (LN CoV-2(−)).

The analysis of surface expression of CD197 (CCR7) and CD45RA within CD4-positive and CD8-positive subpopulations allowed us to follow the phenotypic classification of the T-cell memory compartment: naive T cells (CD197+CD45RA+ cells), central memory T cells (Tcms) (CD197+CD45RA− cells), effector memory T cells (Tems) (CD197−CD45RA− cells), and effector memory re-expressing T cells (Temras) (CD197−CD45RA+ cells). In addition, regulatory CD4+ T cells (Tregs) were identified based on the surface expression of CD25 and CD127 (CD4+ CD25+CD127− cells).

Fifty thousand events corresponding to viable lymphocytes’ light scatter characteristics were acquired from each sample to analyze B-cell subpopulations. First, B cells were identified based on their positivity for CD19 antigen (Supplementary Figure S2A). Then, based on the expression of CD24 and CD38 antigens, we identified transitional B cells (TBs) defined as CD24++CD38++ (Supplementary Figure S2B) and plasmablasts (PBs) as CD24−CD38++CD27+IgD− (Supplementary Figure S2C). In CD38− B cells, we identified double-negative (CD27−IgD−) memory (DNM) B cells, switched memory (SM) B cells (CD27+IgD− cells), non-switched memory (NSM) B cells (CD27+IgD+ cells), and naive B cells (CD27−IgD+ cells) (Supplementary Figure S2D–F).

Twenty thousand events corresponding to viable monocytes’ light scatter characteristics (Supplementary Figure S3A) were acquired from each sample. First, monocytes were identified based on HLA-DR expression (Supplementary Figure S3B) and then classified as classical (CD14++CD16−), intermediate (CD14++CD16+), or non-classical (CD14+CD16++) (Supplementary Figure S3C–E). Next, the percentage of cells expressing TLR2 or TLR4 was identified in each subpopulation.

Fifty thousand events corresponding to viable dendritic cells’ light scatter characteristics (Supplementary Figure S4A) were acquired from each sample. First dendritic cells were based on CD11c, HLA-DR, and CD14 expression (Supplementary Figure S4B,C) and then were classified into the subclasses of conventional DCs as follows: conventional dendritic cell type 1 (cDC1) was identified as Clec9A+ cells (Supplementary Figure S4D), conventional dendritic cell type 2 (cDC2) was classified as CD1c+FcεRIα+ cells (Supplementary Figure S4E), DC2 as CD5+CD163− cells, DC3 as CD5−CD163+ cells (Supplementary Figure S4F), and inflammatory DC3 as CD14+CD163+ cells (Supplementary Figure S4G). CD11c cells with expression of CD80 (Supplementary Figure S4H) and CD86 (Supplementary Figure S4I) were also identified.

Statistical data analysis was conducted using GraphPad 9 statistical software (GraphPad Software, San Diego, CA, USA), version 9. First, the distribution of the examined variables was checked with Kolmogorov–Smirnov and Shapiro–Wilk normality tests. A significance level of *p* < 0.05 was set for all analyses.

## 5. Conclusions

Our results show that SLE patients with renal manifestation present disturbances in the basic immunological parameters of the blood. LN patients primarily have alterations in the proportions of helper and cytotoxic T cells, which are also characterized by high HLA-DR expression. These features appear to be related to SLE, which is a systemic autoimmune disease, regardless of SARS-CoV-2 infection. However, SARS-CoV-2 infection influences B-cell and monocyte compartments. LN patients who have had COVID-19 six months earlier have higher percentages of PB, suggesting readiness for a humoral response to subsequent contact with the virus. They also have higher percentages of classical and intermediate monocytes associated with inflammatory responses.

## Figures and Tables

**Table 1:** Comparison of basic clinical parameters of LN patients and healthy people.

	HC CoV-2(−) (n = 13)	LN CoV-2(+) (n = 17)	LN CoV-2(−) (n = 10)	*p* Value
Age [years]	38.77 ± 9.16	48.82 ± 14.46	41.5 ± 12.37	0.0864
SLE duration [years]	n.a.	16 (2, 37)	14.5 (4, 22)	0.6043
SLICC [score]	n.a.	0 (0, 4)	0.5 (0, 3)	0.9019
SLEDAI [score]	n.a.	6 (2, 16)	10 (0, 26)	0.6750
SLAM-R [score]	n.a.	6 (2, 17)	5 (1, 13)	0.4433
Symptoms [organ affected]	n.a.			
Joints		16 (94.12%)	9 (90%)	
Skin		12 (70.59%)	9 (90%)	
Neurologic		2 (11.76%)	0 (0%)	
Hematologic		15 (88.24%)	8 (80%)	
Renal		17 (100%)	10 (100%)	
LN duration [years]	n.a.	10 (1, 39)	17.5 (6, 38)	0.0929
Treatment	n.a.			
Glucocorticoids		15 (88.24%)	9 (90%)	
Immunosuppressants		11 (54.71%)	8 (80%)	
COVID-19 infection	n.a.		n.a.	
Fever or chills		12 (70.59%)		
Cough		11 (54.71%)		
Shortness of breath or difficulty breathing		2 (11.76%)		
Fatigue		16 (94.12%)		
Muscle or body aches		12 (70.59%)		
New loss of taste or smell		2 (11.76%)		
Sore throat		7 (41.18%)		
Congestion or runny nose		10 (58.82%)		
Nausea or vomiting		2 (11.76%)		
Diarrhea		1 (5.88%)		
Hospitalization		3 (17.65%)		
ESR [mm/h]	n.a.	10.5 (2, 29)	10 (0, 42)	0.0864
eGFR [mL/min/1.73 m^2^]	n.a.	84 (13, 90)	90 (74, 90)	0.0929
Creatinine [mg/dL]	n.a.	0.82 (0.54, 4.17)	0.78 (0.53, 0.85)	0.2231
Anti-dsDNA [IU/mL]	n.a.	212.34 (0, 800)	258.45 (0, 746.73)	0.9794

Data are presented as mean with standard deviation (SD) or medians with minimum and maximum results according to the one-way ANOVA or U Mann–Whitney test. eGFR—estimated glomerular filtration rate; ESR—erythrocyte sedimentation rate; SLICC—Systemic Lupus International Collaborating Clinics classification criteria; SLEDAI—SLE activity was assessed using the SLE Disease Activity Index; SLAM-R—systemic lupus activity measure, revised; anti-dsDNA—anti-double-stranded DNA antibodies; n.a.—not applicable.

**Table 2:** Comparison of T-cell and B-cell subpopulations between LN patients and healthy people.

	HC CoV-2(−)	LN CoV-2(+)	LN CoV-2(−)	*p* Value
CD3^+^ T cells [%]	66.2 (40.9, 86.1)	76.2 (53.2, 90.2)	68.1 (31.3, 92.8)	0.3155
CD3^+^CD4^+^ (Th) cells [%]	**45 (37.7, 70.2)**	31.7 (12, 50.2) *	27.4 (5.7, 77.3) *	**0.0091**
CD3^+^CD8^+^ (Tc) cells [%]	**40.9 (26, 47.3)**	52.5 (32.4, 79) *	56.1 (14.9, 80.1) *	**0.0069**
CD4^+^/CD8^+^ ratio	**1.11 (0.8, 2.7)**	0.63 (0.18, 1.55) *	0.51 (0.08, 5.19) *	**0.0119**
CD4^+^CD28^+^ cells [%]	**99.5 (95.1, 100)**	**95.6 (84.7, 100)**	94.95 (51.7, 99.5) *	**0.0202**
CD4^+^CD69^+^ cells [%]	3.75 (0.59, 9.36)	1.42 (0.44, 8.02)	5.37 (0.51, 18.1)	0.0606
CD4^+^HLA-DR^+^ cells [%]	**3.37 (1.69, 6.12)**	7.27 (2.58, 13.3) *	6.81 (3.57, 18.8) *	**0.0012**
CD8^+^CD28^+^ cells [%]	81.2 (45.6, 96.2)	63.5 (42.8, 95)	49.15 (15.6, 90.2)	0.0639
CD8^+^CD69^+^ cells [%]	2.59 (0.65, 11.4)	2.31 (0.71, 11.7)	3.4 (1.23, 10)	0.4331
CD8^+^HLA-DR^+^ cells [%]	**6.42 (3.78, 31)**	19.6 (2.78, 41.5) *	**21.2 (5.31, 48.7)**	**0.0200**
Naive CD4^+^ T cells [%]	51.5 (7.51, 83)	55.8 (37.6, 77.5)	44 (18, 81.9)	0.3053
CD4^+^ Tcm cells [%]	18.9 (6.4, 75)	17.2 (6.51, 29.2)	15.5 (8.52, 24.7)	0.5662
CD4^+^Tem cells [%]	15.2 (2.79, 30.8)	14.5 (5.62, 31.9)	22.7 (5.46, 40.4)	0.1333
CD4^+^ Temra cells [%]	5.98 (1.46, 21.7)	10.8 (7.32, 18.4)	13.2 (4.08, 48)	0.0726
Naive CD8^+^ T cells [%]	43.4 (16.3, 68.9)	39.1 (11.8, 89.3)	30.55 (14.8, 79.5)	0.6818
CD8^+^ Tcm cells [%]	**2.46 (0.49, 6.1)**	**1.78 (0.54, 5.45)**	0.7 (0.1, 3.38) *	**0.0226**
CD8^+^ Tem cells [%]	5.22 (1.41, 12.5)	3.65 (0.7, 13.7)	2.89 (0.6, 6.65)	0.1186
CD8^+^ Temra cells [%]	43.4 (26.7, 74.8)	56 (9.07, 76.3)	64.6 (13.7, 82.7)	0.4083
Tregs [%]	**6.2 (4.81, 9.07)**	8.68 (5.67, 20.2) *	**8.12 (2.22, 16.5)**	**0.0031**
CD19^+^ B cells [%]	8.01 (3.49, 15.1)	5.34 (2.82, 13)	7.14 (1.06, 15.6)	0.2611
TBs [%]	1.72 (0.03, 3.58)	0.67 (0, 24)	0.44 (0, 8.14)	0.2927
PBs [%]	**0.07 (0, 1.03)**	0.29 (0, 3.53) *	**0.18 (0, 0.6)**	**0.0152**
Naive B cells [%]	64.6 (51.8, 82.4)	74.6 (20, 87.6)	57.5 (4.38, 83.6)	0.2452
DNM B cells [%]	9.25 (4.13, 16.1)	12.3 (3.06, 65.6)	8.43 (4.46, 32.7)	0.7416
NSM B cells [%]	**15.4 (4.3, 29.5)**	2.65 (1.71, 21) **	**13.9 (2.89, 80.6)**	**0.0003**
SM B cells [%]	7.87 (1.79, 22)	9.74 (1.98, 29.6)	7.29 (2.47, 20.2)	0.7412

Data are presented as median with minimum and maximum results according to the Kruskal–Wallis test with a post hoc Dunn test. The results in bold are statistically significant at *p* < 0.05 * vs. healthy control, ** vs. healthy control and LN CoV-2(−). DNM—double-negative memory; TB—transitional B cells; Tc—cytotoxic T cells; Tcm—central memory T cells; Tem—effector memory T cells; Temra—effector memory re-expressing T cells; Th—helper T cells; Tregs—regulatory T cells; PB—plasmablasts; NSM—non-switched memory; SM—switched memory.

**Table 3:** Comparison of monocyte subpopulations between LN patients and healthy people.

	HC CoV-2(−)	LN CoV-2(+)	LN CoV-2(−)	*p* Value
CD14^+^ cells [%]	80.6 (75.7, 89.8)	87.1 (80.3, 95.5) *	91.85 (78.7, 97) *	0.0010
Classical monocytes [%]	69.7 (56,83.4)	79.8 (70.2, 84.5) **	65.35 (45.3, 87.5)	0.0074
TL2^+^ cells [%]	99.9 (99.4, 100)	99.8 (12, 100)	99.6 (99.3, 100) *	0.0295
TL4^+^ cells [%]	99.6 (99, 100)	99.9 (96.8, 100) *	99.7 (99, 100)	0.0341
Intermediate monocytes [%]	3.97 (2.4, 6.62)	5.77 (2.03, 8.74) *	5.44 (3.19, 9.44)	0.0144
TL2^+^ cells [%]	98.7 (79.7, 100)	92.4 (5.26, 99.5) *	94.1 (79.2, 97.5)	0.0202
TL4^+^ cells [%]	100 (98.9, 100)	100 (99, 100)	99.95 (99.2, 100)	0.5787
Non-classical monocytes [%]	7.43 (3.5, 14.7)	5.57 (0.47, 10.7)	4.64 (0.76, 13.8)	0.1839
TL2^+^ cells [%]	66.8 (1.85, 94.5)	24.4 (0.73, 90.1)	55.75 (0.87, 79.5)	0.1057
TL4^+^ cells [%]	99.5 (98.3, 100)	99.8 (90.6, 100)	98.3 (99.7, 100)	0.9063

Data are presented as median with minimum and maximum results according to the Kruskal–Wallis test with a post hoc Dunn test. The results in bold are statistically significant at *p* < 0.05 * vs. healthy control, ** vs. healthy control and LN CoV-2(−).

**Table 4:** Comparison of DC subpopulations between LN patients and healthy people.

	HC CoV-2(−)	LN CoV-2(+)	LN CoV-2(−)	*p* Value
cDC1 [%]	1.92 (0.33, 8.79)	5.78 (0.72, 24.4)	3.73 (2, 25)	0.0585
cDC2 [%]	0.27 (0.1, 5.91)	1.19 (0.14, 16.8)	0.52 (0, 1.88)	0.0982
DC2 [%]	6.67 (0, 12.5)	5.3 (0, 10.5)	0 (0, 11.1)	0.1340
DC3 [%]	62.5 (0, 81.3)	40 (6.67, 93.6)	20.3 (0, 76.4)	0.0497
infl DC3 [%]	40 (0, 86.7)	23.1 (0, 66.7)	13.95 (0, 84.3)	0.1179
CD11c^+^CD80^+^ cells [%]	23.8 (11.6, 39.8)	34.3 (5.2, 63.7)	20.35 (3.61, 78.9)	0.5005
CD11c^+^CD86^+^ cells [%]	97.8 (62.8, 99.6)	97.7 (71.3, 99.4)	95.6 (86.2, 99.7)	0.9127

Data are presented as median with minimum and maximum results according to the Kruskal–Wallis test with a post hoc Dunn test.

**Table 5:** Comparison of serum cytokines between LN patients and healthy people.

	HC CoV-2(−)	LN CoV-2(+)	LN CoV-2(−)	*p* Value
IFN-γ [pg/mL]	3.94 (2.56, 14.33)	3.74 (2.89, 5.31)	4.03 (2.56, 6.17)	0.5066
IL-2 [pg/mL]	3.65 (2.34, 4.88)	3.65 (2.70, 5.60)	4.07 (0, 6.12)	0.5966
IL-4 [pg/mL]	3.53 (1.78, 5.72)	3.40 (2.28, 5.48)	3.68 (1.45, 5.26)	0.8949
IL-6 [pg/mL]	**5.35 (1.91, 7.29)**	7.58 (3.55, 36.01) *	**7.61 (2.54, 15.37)**	**0.0206**
IL-10 [pg/mL]	**1.95 (0.00, 3.22)**	**2.62 (0, 11.59)**	3.50 (0, 10.87) *	**0.0309**
IL-17A [pg/mL]	0 (0, 78.79)	8.27 (0, 66.08)	6.99 (0, 48.05)	0.2179
TNF [pg/mL]	5.55 (0, 6.68)	4.37 (2.48, 42.43)	5.55 (0, 8.93)	0.7973

Data are presented as median with minimum and maximum results according to the Kruskal–Wallis test with a post hoc Dunn test. The results in bold are statistically significant at *p* < 0.05 * vs. healthy control.

## Data Availability

The data presented in this study are available on request from the corresponding author.

## Supplementary Materials

The following supporting information can be downloaded at: https://www.mdpi.com/article/10.3390/ijms25158278/s1.
