# Supplementary material for: Peripheral Blood Mononuclear Cells and Serum Cytokines in Patients with Lupus Nephritis after COVID-19
Source: Int J Mol Sci. 2024 Jul 29;25(15):8278. doi: 10.3390/ijms25158278 (PMC11311954; doi:10.3390/ijms25158278)
Supplement: Supplementary file 1 [file ijms-25-08278-s001.zip › ijms-3109637-supplementary.pdf]

International Journal of
Molecular Sciences

MDPI

Table S1. Comparison of serum Spike Ig and Nucleocapsid between LN patients and healthy people.

	HC CoV-2(−)	LN CoV-2(+)	LN CoV-2(−)	*p* Value
Spike [U/ml]	290480 (19880, 388480)	281880 (5280, 372580)	216080 (39180, 369680)	0.6780
Nucleocapsid [ng/ml]	0 (0, 0.14)	0 (0, 5.05)	0 (0, 5.84)	0.0914

Data are presented as median with minimum and maximum results according to the Kruskal-Wallis test with a post hoc Dunn test.

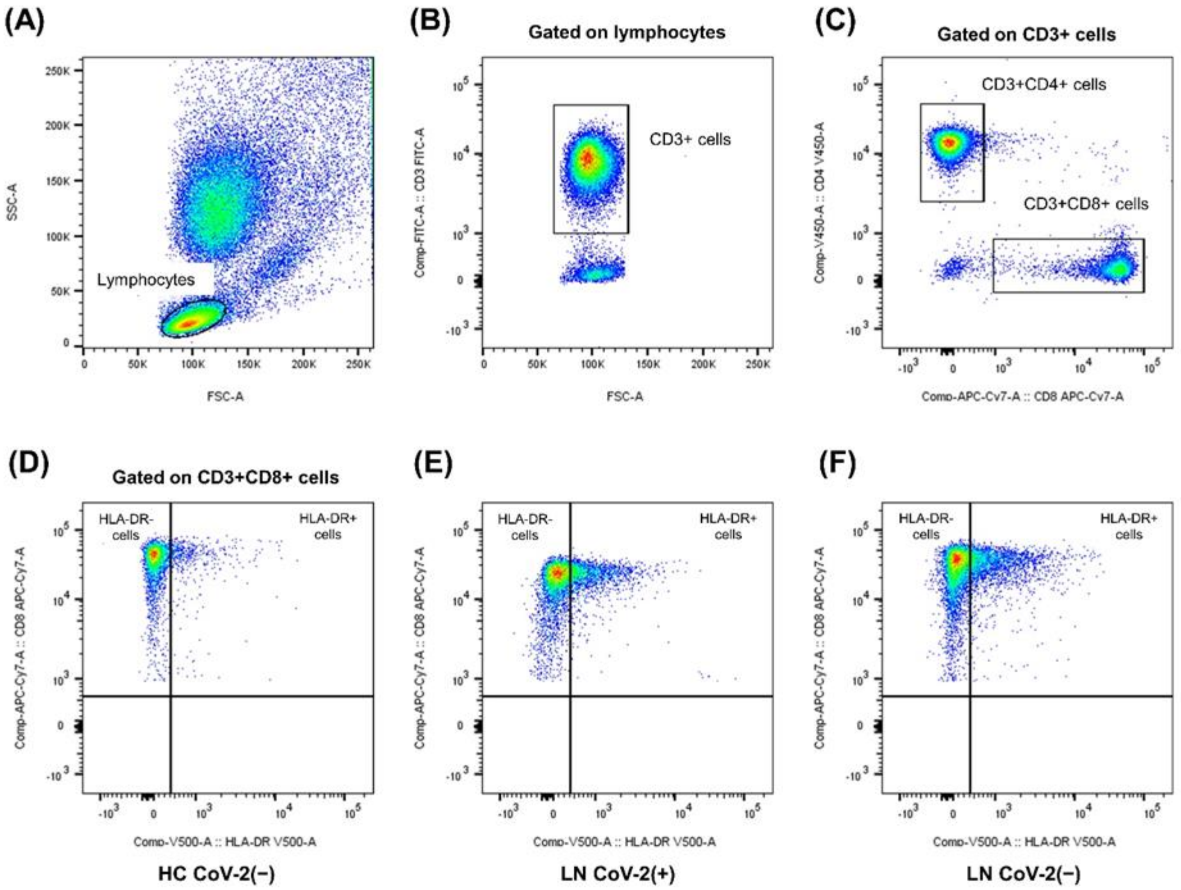

Figure S1. Cytometric analysis of T-cell subpopulations. Lymphocytes were selected based on FSC (forward side scatter) and SSC (side scatter characteristics) (A). Then, T cells were identified based on their positivity for the CD3 antigen (B). Next, helper T cells were identified based on the expression of the CD4 antigen and cytotoxic T cells based on CD8 expression (C). Finally, subpopulations expressing different activation antigens, e.g., the HLA-DR antigen, were identified (D-F). Exemplary dot plots with CD8+HLA-DR+ cells from a healthy donor (HC CoV-2(−)) (D), LN patient six months after SARS-CoV-2 infection (LN CoV-2(+) (E) and from LN patient unexposed to SARS-CoV-2 (LN CoV-2(−)) (F).

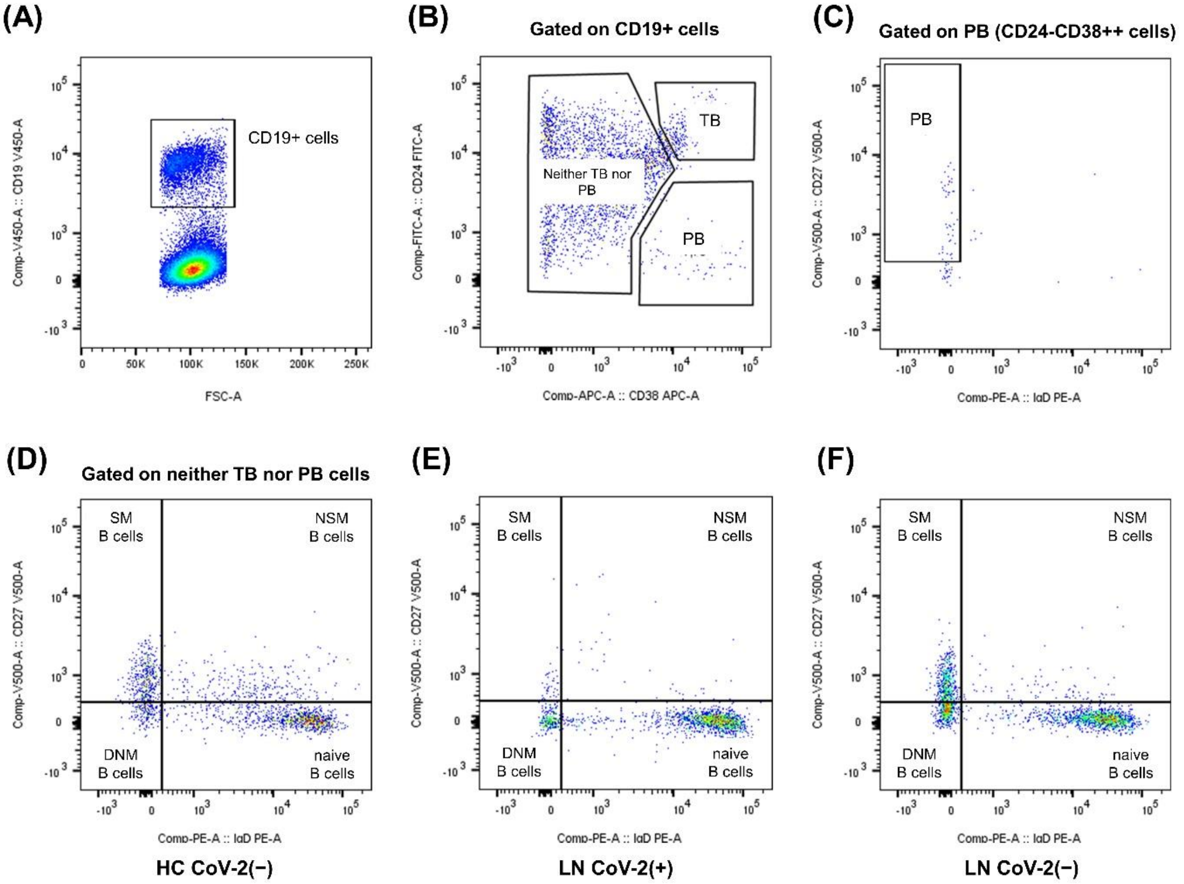

Figure S2. Cytometric analysis of B-cell subpopulations. B cells were identified based on their positivity for the CD19 antigen (A). Based on the expression CD24 and CD38 antigens, transitional B cells (TB) were defined as CD19+CD24++CD38++ (B) and plasmablasts (PB) as CD19+CD24−CD38++CD27+IgD− (C). In B cells CD24−CD38−, double-negative memory (DNM) B cells, switched memory (SM) B cells (CD27+IgD− cells), non-switched memory (NSM) B cells (CD27+IgD+ cells), and naive B cells (CD27−IgD+ cells) (D-F) were identified. Exemplary dot plots with B-cell memory compartment from a healthy donor (HC Co-V-2(−)) (D), LN patient six months after SARS-CoV-2 infection (LN CoV-2(+) (E) and from LN patient unexposed to SARS-CoV-2 (LN CoV-2(−)) (F).

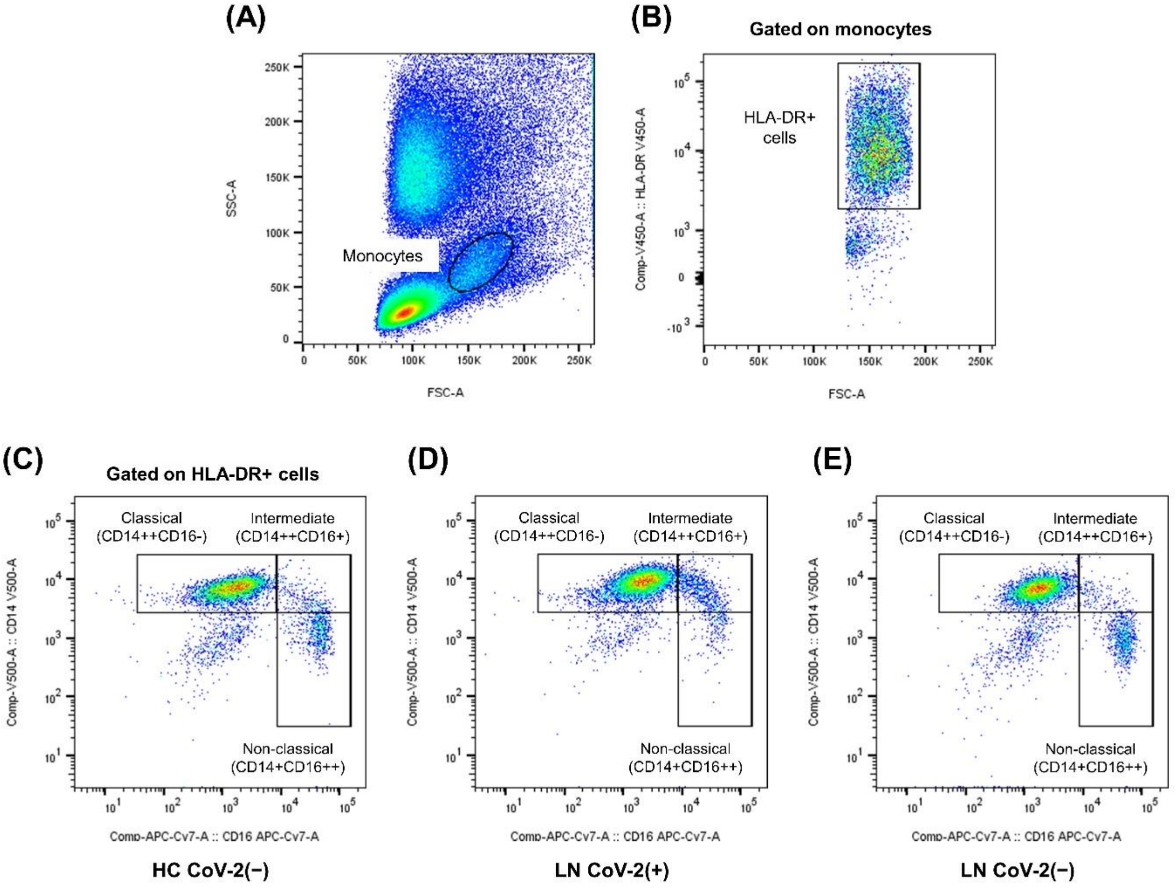

Figure S3. Cytometric analysis of monocyte subpopulations. Monocytes were selected based on FSC (forward side scatter) and SSC (side scatter characteristics) (A). Then, they were identified based on HLA-DR expression (B) and classified as classical (CD14++CD16−), intermediate (CD14++CD16+), or non-classical (CD14+CD16++) (C). Exemplary dot plots with monocyte subpopulations from a healthy donor (HC Co-V-2(−)) (C), LN patient six months after SARS-CoV-2 infection (LN CoV-2(+) (D) and from LN patient unexposed to SARS-CoV-2 (LN CoV-2(−)) (E).

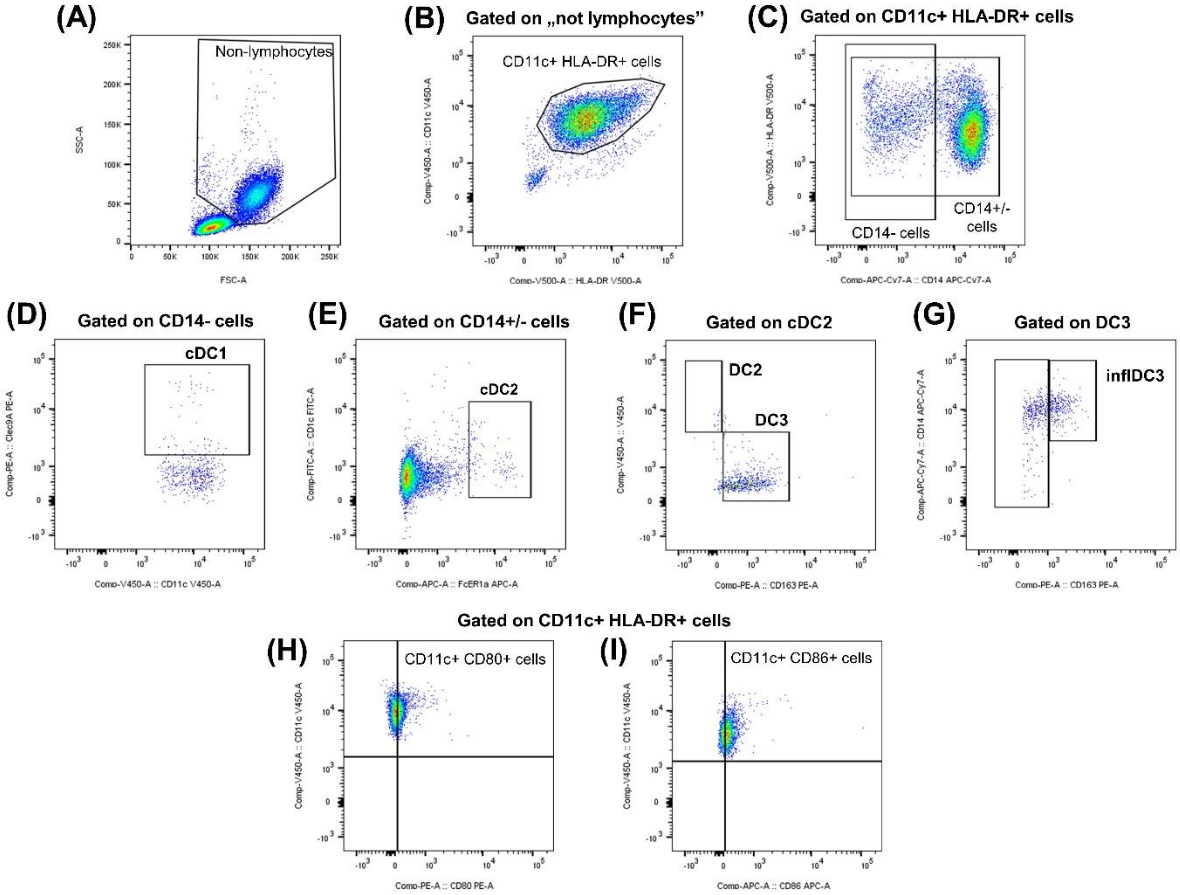

Figure 4. Cytometric analysis of DC subpopulations. Non-lymphocytes were selected based on FSC (forward side scatter) and SSC (side scatter characteristics) (A). Then, dendritic cells were detected based on the CD11c and HLA-DR expression (B). cDC1s (D) were identified as CLEC9A+ from the CD14− (C) fraction of CD11c+HLA-DR+ cells. cDC2 (E) were detected among CD14+/− cells (C) as CD1c+FcεRIα+ cells. Among cDC2, DC2 (CD5+CD163−) and DC3 (CD5−CD163+) were identified (F). Inflammatory DC3s were recognized as CD14+CD163+ cells (G). Mature and activated DCs were identified as CD11c+CD80+ (H) and CD11c+CD86+ (I) cells.
